# SGLT2 inhibitors in the treatment of type 2 cardiorenal syndrome: Focus on renal tubules

**DOI:** 10.3389/fneph.2022.1109321

**Published:** 2023-01-12

**Authors:** Yajiao Wang, Xinxin Mao, Shuqing Shi, Xia Xu, Jiayu Lv, Bingxuan Zhang, Huaqin Wu, Qingqiao Song

**Affiliations:** Guang ‘anmen Hospital, Chinese Academy of Traditional Chinese Medicine, Beijing, China

**Keywords:** SGLT2 inhibitors, cardiorenal syndrome, renal tubules, pathogenesis, renal congestion

## Abstract

The pathogenesis of type 2 cardiorenal syndrome (CRS) is mostly associated with reduced cardiac output, increased central venous pressure (CVP), activation of the renin-angiotensin-aldosterone system (RAAS), inflammation, and oxidative stress. As a drug to treat diabetes, sodium-glucose transporter 2 inhibitor (SGLT2i) has been gradually found to have a protective effect on the heart and kidney and has a certain therapeutic effect on CRS. In the process of chronic heart failure (CHF) leading to chronic renal insufficiency, the renal tubular system, as the main functional part of the kidney, is the first to be damaged, but this damage can be reversed. In this review, we focus on the protective mechanisms of SGLT2i targeting renal tubular in the treatment of CRS, including natriuresis and diuresis to relieve renal congestion, attenuate renal tubular fibrosis, improve energy metabolism of renal tubular, and slow tubular inflammation and oxidative stress. This may have beneficial effects on the treatment of CRS and is a direction for future research.

## 1 Introduction

In 2008, Prof. Ronco (1) defined cardiorenal syndrome (CRS) as a disease of the heart and kidneys in which acute or chronic dysfunction in one organ may lead to acute or chronic dysfunction in the other. There are 5 types of CRS, of which chronic renal insufficiency due to chronic heart failure (CHF) was defined as type 2 CRS. Studies have shown that approximately 40-50% of patients with CHF also have chronic kidney disease(CKD) (2), and renal insufficiency is one of the most important risk factors for predicting mortality and poor prognosis in patients with heart failure (HF), with the risk of death more than doubling in the presence of CKD (3, 4). Clinical treatment of CRS is intractable and expensive, which imposes a heavy burden on society and families. Therefore, effectively delaying the occurrence and development of type 2 CRS is an important measure to reduce mortality and medical burden.

**Figure 1:**
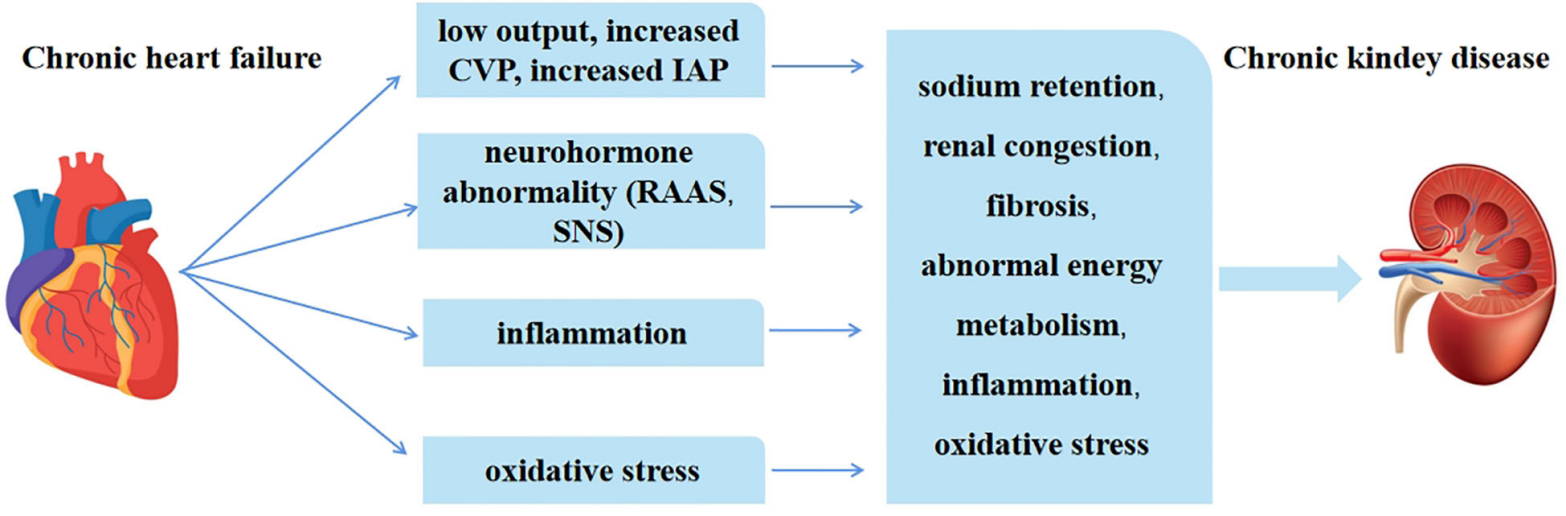
The main pathological mechanism of type 2 CRS.

There are various hypotheses for the pathogenesis of type 2 CRS, including reduced cardiac output, increased central venous pressure (CVP) and intra-abdominal pressure, activation of the renin-angiotensin-aldosterone system (RAAS), inflammation and oxidative stress, etc. (5) (**Figure 1**). Renal dysfunction is mainly associated with hemodynamic alterations and neurohormonal activation. The current main treatments for CRS include decongestive therapy and neurohormonal modulation of vasodilators and so on (6). However, how to effectively delay or even reverse the occurrence and development of CRS is crucial.

The main task of the renal tubular is to regulate sodium and volume homeostasis. A healthy kidney needs to filter 180 liters of 1.5 kg sodium chloride ultrafiltrate every day, but only less than 1% of sodium chloride is discharged in the end (7), and Small lesions in the renal tubules will affect the volume and electrolyte homeostasis. Before the decrease of glomerular filtration rate (GRF) and the appearance of proteinuria in CHF patients, the markers of renal tubular injury, such as NGAL and KIM-1, have been significantly increased (8). The early swelling and necrosis of renal tubular injury in type 2 CRS rats indicate that renal tubular injury is the starting link of CRS (9). Renal tubule injury has been proven to be an important factor in the development of renal insufficiency and glomerular injury and is related to the severity and poor prognosis of CHF (10). Therefore, renal tubule injury is an important pathological process of early renal function injury in CHF, and improving renal tubule injury is of great significance in delaying the development of CHF to CRS.

**Figure 2:**
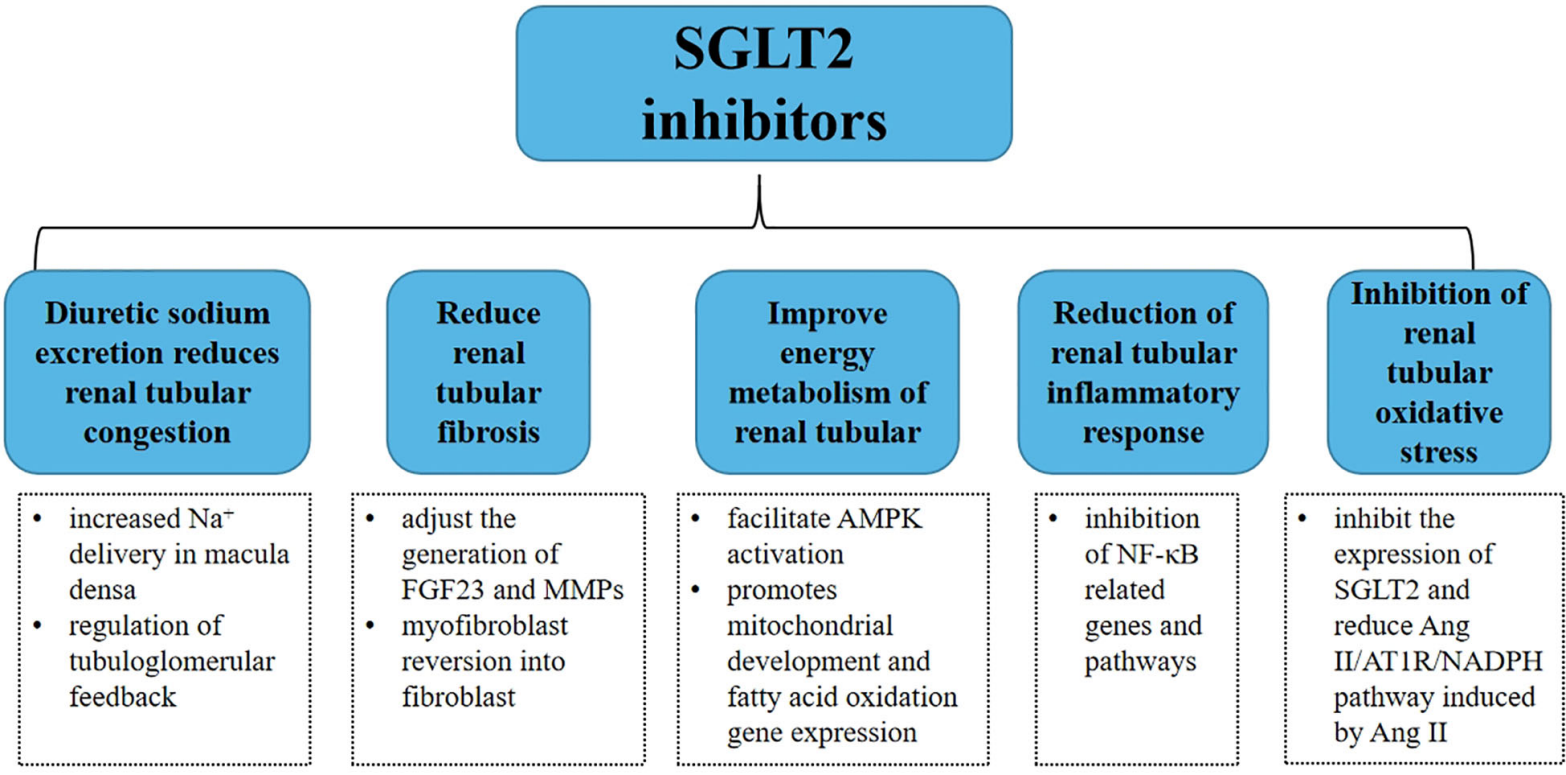
Summary of potential mechanisms of SGLT2 inhibitors in renal tubule therapy for type 2 CRS.

Sodium-glucose cotransporters (SGLTs) are a family of six protein isoforms, including SGLT1, SGLT2, SGLT3, SGLT4, SGLT5, and SGLT6 (11), which can mediate the transport of glucose, ions, osmotic substances, vitamins, and amino acids. Among them, SGLT2 almost only exists in the lumen of epithelial cells in the first and second segments of proximal tubules (12). As a phlorizin derivative, sodium-glucose transporter 2 inhibitor (SGLT2i) mainly acts on renal tubules. In recent years, it has been gradually found that SGLT2i can treat heart failure, reduce the hospitalization rate and mortality due to heart failure, and has a renal protective effect (13). As shown in **Figure 2**, this review focuses on renal tubular and illustrates the therapeutic effect of SGLT2i on CRS.

## 2 Diuretic sodium excretion reduces renal tubular congestion

### 2.1 Hemodynamic changes in type 2 CRS

Renal hemodynamic alterations in CHF patients mainly include decreased blood flow and increased venous pressure. In 2021, Husain et al. (14) defined this renal lesion associated with venous congestion as well as decreased renal perfusion as congestive nephropathy.

The kidney is dependent on contraction and relaxation of the heart for adequate transrenal pressure gradients to maintain renal blood flow. It receives approximately 20% of cardiac output, and a decrease in CHF cardiac output leads to a decrease in effective renal circulating blood volume and renal hemodynamic alterations. At this point, this will cause prerenal renal injury, and activation of the RAAS, triggering increased aldosterone secretion and water-sodium retention. Then increased sodium and water reabsorption by the renal tubules, fluid retention, and deterioration of cardiac pumping capacity create a vicious cycle. The purpose of RAAS activation is to keep renal perfusion and maintain effective plasma volume through renal tubular reabsorption. But the reabsorbed water and sodium will eventually lead to oliguria and worsening renal congestion, which further exacerbates renal hemodynamic abnormalities and renal burden, and the vasoconstrictive effect of renin-angiotensin may cause a further decrease in renal blood flow.

Central venous pressure (CVP) is an important indicator to evaluate venous congestion. The heart’s pumping function is damaged, and blood cannot flow back smoothly and accumulate in the vein, in addition to the renal venous pressure itself, the central venous pressure also increases. The resistance of venous vessels is very small. The increase of central venous pressure can directly increase the renal venous pressure, reduce the renal blood flow, and cause the increase of renal interstitial pressure, which is further transmitted to the renal tubules to resist the hydrostatic pressure in the glomerulus, which will reduce the glomerular filtration rate (15). And with the increase of renal venous pressure, the concentration of angiotensin II will also increase (16), which undoubtedly aggravates kidney disease. CVP is a powerful hemodynamic monitoring index for the deterioration of renal function. Many studies show that increased CVP is associated with decreased kidney function and increased mortality. In 2022, a retrospective observational cohort study divided 61,532 patients in Intensive Care Unit into four groups based on mean CVP 7 days before admission and recorded their creatinine and urine output. This study found that patients with higher mean CVP had lower urine output, higher creatinine, and lower eGFR. Logistic regression results suggested that the incidence of AKI is higher in patients with a CVP higher than the mean CVP, ranging from OR = 1.63 (95% CI:1.44-1.85) to OR=5.18 (95% CI: 4.37-6.14) after adjustment, mean CVP remains a significant predictor of AKI (17, 18). Early implementation of CVP testing allows for better fluid management (19).

Elevated intra-abdominal pressure (IAP) reduces blood flow to the kidney and also compresses the renal veins and ureters, causing renal venous congestion. Acute tubular injury may develop further with increased IAP (20). IAP is significantly associated with renal failure and may rise mortality. A study looking at the relationship between IAP and indicators of renal function as well as all-cause mortality by measuring IAP in 43 patients with acute decompensated heart failure found that higher IAP at admission was associated with poorer baseline renal function and that a persistent IAP above 12 mmHg within 72 hours was associated with longer length of hospital stay and higher 1-year all-cause mortality (45%, log-rank test = 0.041) (21, 22).

Studies have shown that patients with higher hospitalization rates of HF, (reflecting a persistent congestive state) are associated with a long-term decrease in glomerular filtration rate, that decongestive therapy improves overall outcomes (23, 24), and that volume overload is associated with progressive renal disease (25). Fortunately, congestive nephropathy appears to be reversible in the early stages. There are animal studies have demonstrated that elevated renal venous pressure leads to decreased renal artery blood flow and glomerular filtration, increased plasma renin activity, serum aldosterone, and urinary protein, and that congestion-induced renal dysfunction can partially or completely be eliminated when RVP is reduced to normal levels (26). In patients with acute heart failure (AHF), reducing already elevated IAP by decongestive therapy can improve renal function (27). Therefore, early achievement of decongestive therapy to effectively relieve renal congestion is an important step in delaying the development of type 2 CRS.

### 2.2 SGLT2i restores tubuloglomerular feedback

For a state of renal congestion, it is important to both decongest and have no residual volume overload and to maintain effective perfusion of the kidney. A commonly used clinical drug to reduce congestion is diuretics. Among diuretics, thiazide and loop diuretics are used as first-line drugs for decongestion. The most significant deficiency is diuretic resistance due to multiple mechanisms, such as impaired renal function, reduced cardiac output resulting in decreased delivery of diuretics to the kidney, inadequate diuretic doses, or insufficient substrate at the renal tubules (28). In patients with HF, the dose-response curve for diuretics shifts to the lower right, so that higher doses of diuretics can be effective in HF, but high doses of diuretics are associated with worsening renal function (29), and long-term use of loop diuretics leads to renal remodeling, hypertrophy, and hyperplasia of distal renal tubules. Renal insufficiency decreases diuretic excretion into the tubular lumen, while urinary sodium excretion in CKD is reduced by decreased sodium filtration (30, 31). Ultrafiltration is another treatment to reduce renal congestion but has been associated with a higher incidence of adverse events in clinical trials (32, 33).

The state of renal congestion is closely related to renal tubular diseases. Tubuloglomerular feedback (TGF) is a response that occurs between the renal tubules and the small arteries. It is primarily a physiological response triggered by 15-20 specific tubular epithelial cell plaques-macula densa located in the distal tubules (34). The macula densa senses the sodium concentration of renal tubules to regulate the contraction of the afferent arterioles, affect the renal blood flow, and change the glomerular filtration rate. The reabsorption of sodium in the proximal tubules can determine the concentration of liquid solute reaching the macula densa and the activation of TGF. When the volume in the physiological state is insufficient, the content of Na^+^ transported to the macula densa decreases, and the adenosine produced by the macula densa decreases, which then makes the entering artery relax, and maintains the stability of renal blood flow and glomerular filtration rate. Under pathological conditions, when the concentration of sodium ions flowing through the macula densa increases, the entering arterioles contract, the renal blood flow decreases, and the glomerular filtration rate decreases. When the kidney is congested, the renal perfusion is reduced, and the compensatory reabsorption of renal tubules is increased to maintain the plasma volume. At this time, with the increase of sodium reabsorption, the content of Na^+^ in the distal convoluted tubule decreased, and the Na^+^ transported to the macula densa decreased. The small artery entering the glomerulus relaxed, and the pressure in the glomerular sac increased, resulting in a hyperfiltration state, which increased the burden on the kidney, and the tubular bulb fed back TGF inactivated (35). Therefore, restoring the normality of TGF under renal congestion is an important direction to delay the progress of renal injury.

One of the main assumptions of SGLT2 inhibitors for renal protection is that they can improve renal hemodynamics, reduce renal tubular reabsorption, increase the concentration of sodium in the distal nephron, sodium transport, and dense plaque transport, and restore TGF is the core of SGLT2i’s renal protection (36). After SGLT2i was used, it blocked Na^+^ reabsorption in renal tubules, increased Na^+^ content in distal convoluted tubules, increased Na^+^ in macula densa, contracted the small artery entering the bulb, decreased the pressure in the capsule, recovered the ultrafiltration phenomenon, and repaired the tube bulb feedback. Furthermore, the glomerular hemodynamics is changed through the tubular bulb feedback mechanism, leading to the decrease of glomerular internal pressure and the reduction of GFR, which is transient and will not cause permanent damage to renal function (37).

Borges-Júnior FA et al. (38) used a rat model of HF due to myocardial infarction and a sham-operated group of rats, and each of the two groups of rats was further divided into two groups, the control group and the group treated with Empagliflozin. After saline challenge (intraperitoneal injection of 0.9% NaCl of a certain volume), the diuretic and natriuretic responses in Empagliflozin-treated HF rats were similar to those in sham-operated rats and higher than those in untreated HF rats. The hematocrit levels of untreated HF rats were lower than those of sham-operated rats, Empagliflozin treatment restored the hematocrit of HF rats to levels similar to those of sham-operated rats, restored the blood volume of HF rats, and improved renal handling of sodium and water, as evidenced by normalization of hematocrit and serum brain natriuretic peptide (BNP) levels. This suggests that SGLT2i can improve hemodynamics through sodium excretion and diuresis.

### 2.3 SGLT2i inhibits NHE3 transport activity

Sodium-hydrogen exchange proteins (NHE) are a family of ion transport pump proteins that regulate the mutual exchange between extracellular Na+ and intracellular H^+^ to transport Na^+^ into the cell and H^+^ out of the cell, maintaining acid-base and electrolyte balance inside and outside the cell. NHE has different isoforms, and among all Na^+^ transport proteins and co-transport proteins, NHE3 is the most critical Na^+^ transporter protein responsible for Na^+^ reabsorption in the kidney. NHE3 is mainly located in the proximal tubule as well as in the crude branching segments of the kidney and small intestine. In the kidney, NHE3 is expressed mainly along the parietal membrane of the proximal tubule. It absorbs most of the sodium originating from the intestine and reabsorbs more than 50% of the filtered Na^+^ in the renal tubules (39). Angiotensin II (AngII) is the main stimulator of renal NHE. In the NHE3 knockout mouse model, mice show significant diarrhea and hyponatremia. Compared to control mice, model mice showed significantly increased fluid intake and significantly higher erythrocyte-specific volume, suggesting dehydration and volume deficit (40). It can be seen that NHE3 is essential to maintain sodium and body fluid homeostasis.

Due to venous congestion and hypoperfusion, enterocytes and tubular epithelial cells are hypoxic, resulting in elevated expression of NHE3, increased intestinal sodium absorption, and proximal tubular reabsorption, thus leading to TGF inactivation, fluid retention, and exacerbate cardiac burden and renal damage. Clinical trials have confirmed that Tenapanor, an NHE3 inhibitor, targets intestinal NHE3 therapeutically. It reduces intestinal sodium absorption, significantly increases fecal sodium content, and is therapeutic for patients with fluid overload HF (41). Sodium reabsorption by NHE3 in the renal tubules is greater than 50%. So, in addition to gastrointestinal NHE3 inhibition, renal NHE3 inhibition could also be a direction of study in HF patients.

**Figure 3:**
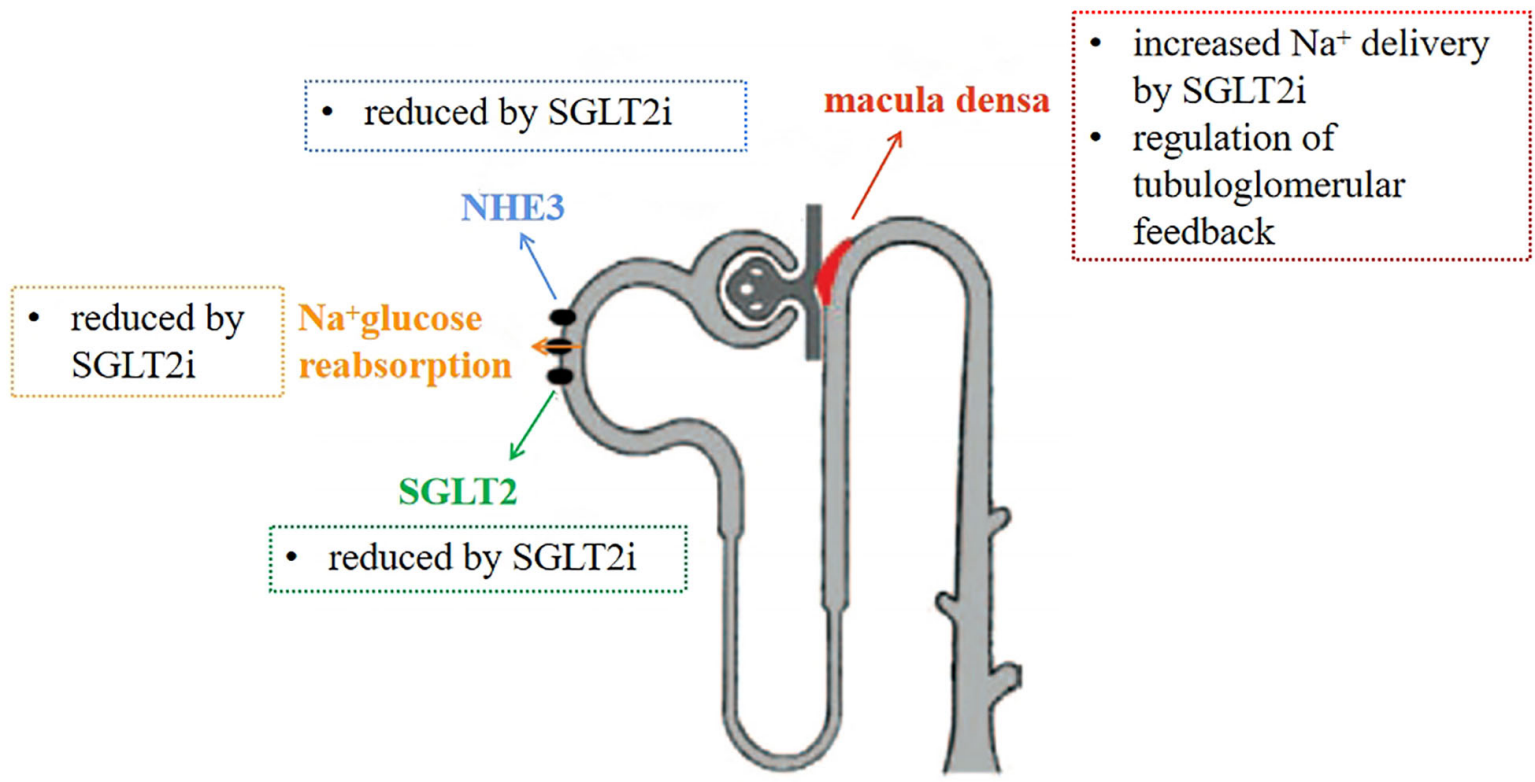
Mechanism of diuretic sodium excretion of SGLT2 inhibitors. Inhibit NHE3 and SGLT2 proteins, reduce the reabsorption of sodium by renal tubules, increase the concentration of sodium ions in macula densa, and restore tubuloglomerular feedback.

SGLT2 and NHE3 co-exist in the renal tubular apical membrane. Borges-Júnior FA et al. (38)evaluated SGLT2 and NHE3 expression by immunoblotting and quantitative RT-PCR after 4 weeks. Proximal tubule NHE3 activity was measured by fixed *in vivo* microperfusion. The results showed that proximal tubule NHE3 activity was higher in HF rats than in sham-operated rats, while EMPA treatment significantly reduced NHE3 activity. It is suggested that SGLT2i may act on NHE3 to achieve diuretic and sodium-removal effects by inhibiting its expression, and improving hemodynamics, reducing renal congestion, thus further preventing renal pathology. This was also confirmed by mathematical model predictions (42). If the direct inhibition of SGLT2 is the only result of dapagliflozin treatment, the Na^+^reabsorption at the distal end of the common renal tubule will increase slightly, but this behavior has not been observed clinically, so this situation cannot represent the full effect of SGLT inhibition. Finally, it is predicted that NHE3 is necessary for SGLT2i’s natriuretic effect. NHE3 can reabsorb 50% of the sodium from the renal tubules, so it is necessary to affect NHE3 to achieve sodium-removal efficacy in the clinic. Long-term application of Empagliflozin enhances the phosphorylation of NHE3 in Akita diabetes mice and reduces the activity of NHE3 (43). It is known that when SGLT2i inhibits NHE3 protein activity, sodium reabsorption by proximal tubules is inhibited, solute delivery to the macula densa is increased, and TGF activity increases, thereby restoring renal hemodynamic function (44). SGLT2i can also reduce the expression and activity of NHE in the kidney through the inhibition of RASS, thereby reducing ultrafiltration and the accompanying renal disease, playing a renal protective role (**Figure 3**). At present, no evidence of plasma volume contraction or renal function impairment has been found, which may prevent plasma volume depletion and subsequent low perfusion.

## 3 Reduce renal tubular fibrosis

### 3.1 Renal tubular fibrosis in type 2 CRS

Hemodynamic alterations and over-activation of the RAAS system occur in patients with CHF. Hemodynamic alterations are early pathological changes in the deterioration of renal function. If renal tissue hypoperfusion and renal congestion develop further, renal tubular atrophy and tubulointerstitial damage as well as renal fibrosis will be triggered (45, 46). Ang II leads to renal hypoxia, vasoconstriction, intra-glomerular hypertension, glomerulosclerosis, tubulointerstitial fibrosis, and proteinuria after over-activation of RAAS.

Fibrosis is the result of cell deposition. Necrotizing apoptosis of renal tubular epithelial cells mediates tubulointerstitial fibrosis in patients with CKD, which is a common pathological manifestation of renal lesions, and the progression of fibrosis results in progressive loss of renal function. Tubulointerstitial fibrosis is accompanied by tubular cell basement membrane thickening, hyperplasia, and hypertrophy, which is ultimately associated with renal failure. The main pathological process is the progressive loss of renal tubular epithelial cells, and excessive deposition of extracellular matrix, resulting in the destruction of renal parenchyma, progressive tubulointerstitial fibrosis, and progressive loss of renal function (47). Compared with the changes in glomerular histology, tubulointerstitial fibrosis is more closely related to the deterioration of renal function. Renal fibrosis can magnify renal damage and accelerate nephron death, which is one of the pathological mechanisms of CRS formation.

Fibroblasts located near the proximal tubule of the kidney produce erythropoietin under physiological conditions, but the selective injury to the proximal tubule epithelial cells under hypoxic conditions induces fibroblast differentiation into myofibroblasts, and these transformed fibroblasts are unable to produce erythropoietin, but actively produce fibrotic molecules (48). In CKD, fibroblast conversion is the main source of fibrosis, but this conversion is reversible, and myofibroblasts can revert to fibroblasts as well as regain their original physiological properties when proximal tubule epithelial cells injury subsides. The prognosis of renal insufficiency is mainly related to the degree of tubular interstitial fibrosis rather than glomerular injury (49). Therefore, early intervention of tubular fibrosis can reverse renal lesions and improve CRS.

### 3.2 SGLT2i regulates fibrosis related proteins

Myofibroblasts are contractile and alter the renal structure, producing a variety of excreted factors such as fibroblast growth factor 23 (FGF23) (50). FGF23 plays an important role in the pathogenesis of CRS, not only as a sensitive marker of renal processing of phosphate abnormalities but also as a close link to renal interstitial fibrosis. FGF23 is elevated in the early stages of nephritic damage, and as the renal disease progresses, plasma FGF23 levels continue to increase, and high levels of FGF23 cause left ventricular hypertrophy, renal function deteriorated, and increased mortality rate (51). Up-regulation of FGF23 may be achieved by activating the TGF-β related pathway to enhance myofibroblast activation and fibrosis. Moreover, FGF23 is not only expressed in cardiomyocytes and fibroblasts, but also promotes myocardial fibrosis in mice with ischemia/reperfusion injury (52, 53). Huixin Hao et al. (54)performed surgery on mice to cause myocardial infarction and formed a mouse model of CRS after 12 weeks. After that, They found that cardiac remodeling, myocardial fibrosis, and cardiac dysfunction accompanied by glomerular injury and tubulointerstitial fibrosis increased, resulting in type 2 CRS. Mice with CRS showed increased renal FGF23 protein levels as well as cardiorenal fibrosis. It is suggested that abnormal elevation of FGF23 is involved in the development of type 2 CRS and the mechanism of involvement is related to renal fibrosis. Matrix metalloproteinases (MMPs) are involved in collagen metabolism and also in the progression of renal fibrosis. SGLT2i can regulate the production of FGF23 as well as MMPs (55, 56), thus delaying renal interstitial fibrosis. Not only that, but SGLT2 inhibitors can also alleviate the hypoxic environment of renal tubular by inhibiting Na^+^/K^+^ pumps to reduce ATP consumption and alleviate metabolic stress in proximal tubule epithelial cells, which facilitates the reversal of myofibroblasts into fibroblasts and attenuates renal tubular fibrosis (57).

Kojima N et al. (58) used luseogliflozin for the long-term treatment of T2DN rats and histologically evaluated the kidney at the end of the treatment. They found that luseogliflozin significantly reduced the extent of renal fibrosis and protein tubular. In comparison with the efficacy of Canagliflozin and glimepiride in the treatment of diabetes, during the 2-year follow-up period, 300 mg/day of Canagliflozin reduced plasma levels of matrix metalloproteinase 7 (24.9%; *p*=0.011) and proteinuria by 35.7% (95% CI 20.1, 48.3; *p*<0.001) compared with glimepiride (56).

## 4 Improve energy metabolism of renal tubular

As the main functional part of the kidney, renal tubules are involved in renal reabsorption, multiple regulation, endocrine, and other functions, which require large amounts of energy and are susceptible to damage from various stimuli, thus worsening renal function. Renal tubular epithelial cells contain a large number of mitochondria, which reabsorb about 60-70% of Na^+^. This process requires the participation of NHE3, SGLT2, and other NA ion transport proteins, Na^+^ is excreted through an energy-dependent Na/K-ATPase located on the basolateral membrane, Na/K-ATPase is the driving force for Na^+^ reabsorption (59). In CHF patients, due to the activation of RAAS, increased secretion of aldosterone as well as water and sodium reabsorption, which exacerbates the energy burden on the renal tubules and consumes more oxygen. However the renal tubules are more sensitive to hypoxia than the glomeruli, so it is easy to cause renal tubular damage and necrosis. But, hypoxic renal tubular damage is immediate, which can be reversed by reducing the workload of oxygen consumption and restoring mitochondrial structure and function.

Mitochondria pass through fatty acids β- Oxidation maintains the energy supply. Renal proximal tubule cells containing a large number of mitochondria rely on fatty acid oxidation as the energy source. Tubular cells are vulnerable to the influence of mitochondrial fatty acid oxidation disorder. The mitochondrial fatty acid oxidation disorder leads to proximal tubule energy failure, while excessive fatty acid induces the intracellular accumulation of free fatty acids and triglycerides, thus inducing the production of Reactive oxygen species (ROS) in mitochondria (60). High-fat diet mice exhibited tubular vacuole formation, dilatation, and epithelial cell detachment. The ultrastructure showed round or fragmented renal tubular mitochondria, and the inner membrane was also disrupted. After 16 weeks of feeding with ipragliflozin, it reversed the damage to a normal state, restored the expression of optic atrophy factor 1 (Opa1) and mitofusion 2 (Mfn2), and independently of weight loss or hypoglycemic effects, which suggests that SGLT2 inhibition may act directly on renal tubular cells and protect them from mitochondrial damage caused by metabolic injury (61). SGLT2i excrete human glucose into the urine, which leads to increased glucagon levels, decreased insulin secretion, conversion of energy metabolic substrates from glucose to fat, and increased cellular fatty acid oxidation to maintain energy supply. Canagliflozin can improve fatty acid oxidation related to gene CPD1b and mitochondrial biogenesis and functional gene PGC-1 α, Nrf1 expression, suggesting that cagelin can improve mitochondrial fatty acid oxidation and mitochondrial biosynthesis and function (62).

Activated protein kinase (AMPK) participates in mitochondrial biogenesis by activating PGC-1α, and AMPK activation restores energy homeostasis by promoting catabolic pathways (including fatty acid oxidation) and inhibiting anabolic pathways (including fatty acid synthesis). Empagliflozin may promote AMPK activation by regulating cellular adenosine monophosphate (AMP)/adenosine triphosphates (ATP) content (63). Canagliflozin may also significantly increase AMPK expression in HEK-239 cells by increasing cellular AMP or Adenosine Diphosphates (ADP) levels, and the ADP/ATP ratio increases with increasing concentrations, during this process, oxygen consumption decreased somewhat (64).

SGLT2i reduces the reabsorption of filtered sodium and glucose in the proximal tubules, and reduces the workload of oxygen consumption and transportation of renal tubular cells. Less oxygen pressure helps to improve the integrity of renal tubular cells, alleviate the damage of proximal tubules caused by hypoxia, and promote cell integrity. Moreover, SGLT2i can regulate renal tubular energy metabolism by promoting the expression of mitochondria, function-related genes, and fatty acid oxidation-related genes.

## 5 Reduction of renal tubular inflammatory response

### 5.1 Inflammatory response in type 2 CRS

Both CHF and CKD are chronic inflammatory states with elevated levels of circulating inflammatory mediators, and inflammation is a key link in the cardiorenal interaction. Eunjung Cho et al. (65) induced the myocardial infarction model by ligating the left coronary artery, and established the volume failure model by low salt diet and furosemide injection. At the 4th and 8th weeks after myocardial infarction, obvious renal interstitial fibrosis occurred, and CKD gradually occurred. In this process, macrophage infiltration and increased expression of inflammatory cytokines (TNF-α、IL-6) were accompanied, indicating that inflammation played an important role in the pathogenesis of type 2 CRS. Inflammatory factors in CRS patients increased significantly and gradually damaged renal tubular cells. Proteinuria in CRS patients is a sign of renal injury, and proteinuria also has an inflammatory effect on renal tubules. Inflammation is a predictor of mortality and disease severity in patients with heart failure and is also a predictor of mortality in patients with chronic kidney disease (66).

Hyperactivation of neurohormones in CRS is a major biological source of inflammation. Activation of RAAS and Sympathetic nervous system (SNS) promotes inflammatory responses in the heart and kidney. Ang II will increase the activation of NF-κB. NF-κB can regulate a variety of proinflammatory factors, increase the expression of IL-6, and TNF-αin myocardial cells, and renal tubular cells, and is one of the sources of cardionephritis (67, 68). The inflammatory factors produced by cardiac myocytes can not only damage the heart itself but also cause damage to other organs. Renal tubular epithelial cells play an important role in the transmission and release of inflammatory mediators and become the main part of cell damage. Interstitial inflammatory reaction stimulates renal tubule injury. The action of lipopolysaccharide and Toll like receptors located in renal tubule skin cells will induce the release of inflammatory factors. The production and release of inflammatory mediators will change and affect the regulation of renal microcirculation and perfusion distribution, resulting in decreased medullary blood flow, which will adversely affect renal tubule function. Animal studies show that (69), β- Adrenaline activation increases the mRNA expression of proinflammatory cytokine TNF-α, IL-6 in cardiomyocytes. In this process, oxidative stress of renal tubular epithelial cells is a common pathway for cell dysfunction, tissue damage, and organ failure. With further study of heart and kidney diseases, it is found that venous congestion and volume overload can promote inflammation. Organs are forced to play a role under significantly increased venous and interstitial pressure. The effects of vascular stretching and tissue congestion may promote additional inflammation. For example, after the edematous gastrointestinal tract and peripheral tissues are exposed to increased interstitial pressure, they will stimulate the absorption of intestinal endotoxin, peripheral synthesis, and release of inflammatory mediators. This inflammation further damage the functions and structures of important organs such as the heart, vascular system, and kidney. Endotoxin acts as an inflammatory stimulus. The level of endotoxin in CKD patients with fluid overload is higher than that in CKD patients without fluid load (70). It suggests that the source of CRS inflammation may be the joint action of the above mechanisms.

### 5.2 SGLT2i inhibits NF-κB related pathway

**Figure 4:**
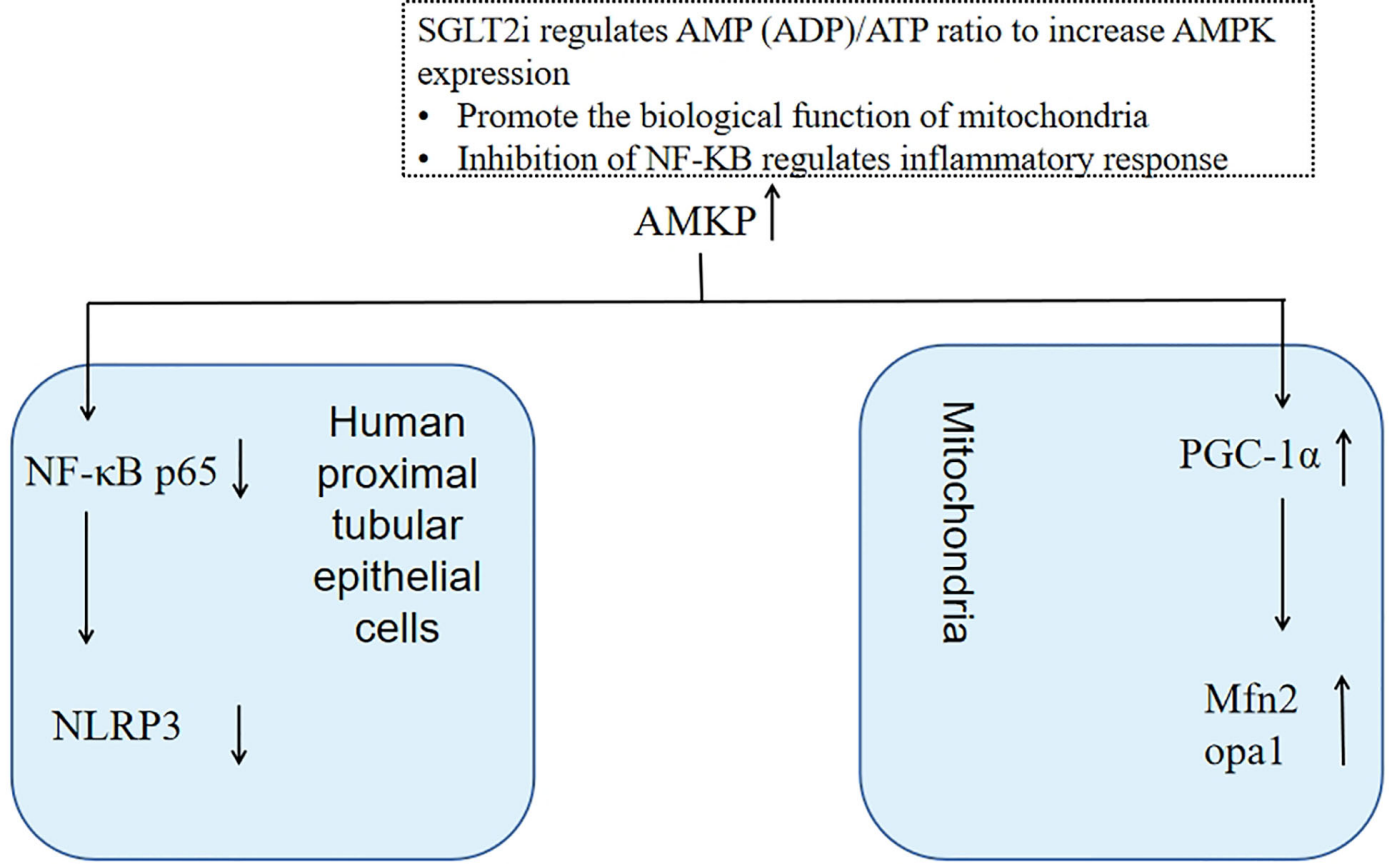
SGLT2i increases AMPK expression by regulating AMP(ADP)/ATP ratio, which can not only regulate mitochondrial function to maintain renal tubular energy metabolism but also inhibit NF-kB to reduce inflammatory reactions.

NLRP3 activation may be involved in multiple inflammatory or stress pathways, such as the NF-κB signaling pathway. AMPK activation may inhibit the protein modification of NF-κB p65. Dapagliflozin reduces high glucose induced inflammasome activation and expression of TNFα by inhibiting NF-κB p65 nuclear translocation, and this effect is lost after knockdown of AMPK, suggesting that the effect of dapagliflozin on inflammation possibly through AMPK activation to inhibit nuclear NF-κB p65. AMPK/NF-κB p65/NLRP3 signal transduction pathway may be a potential mechanism for dapagliflozin to play an anti-inflammatory role in human renal proximal tubular cells (71)(**Figure 4**).

An analysis of the results of a randomized crossover trial found that dapagliflozin reduced albuminuria and decreased the excretion of KIM, a marker of renal tubular injury, and IL-6, an inflammatory factor. The effect of dapagliflozin in reducing proteinuria may be due to reduced intra-glomerular pressure or tubular cell injury (72). High mobility group box 1 (HMGB1) can increase the expression and secretion of inflammatory and oxidative stress markers such as superoxide dismutase (SOD), malondialdehyde, and fibronectin. While dapagliflozin can reverse these effects. Culturing human proximal tubular epithelial cells revealed that dagliflozin may mediate the inhibition of HMGB1-RAGE-NF-κB signaling pathway to attenuate the expression of inflammatory markers, thereby delaying renal injury (73).

## 6 Inhibition of renal tubular oxidative stress

Oxidative stress is an imbalance between oxidants and antioxidants that allows excessive accumulation of the former eventually leading to cellular damage (74). ROS are mainly produced in mitochondria and oxidative stress occurs when the level of ROS increases beyond the body’s antioxidant capacity, leading to cellular damage and endothelial dysfunction. Activation of RAAS leads to apoptosis and hypertrophy in the heart and kidney, impairing mitochondrial function and exacerbating mitochondrial generated oxidative stress. Ang II mediates oxidative stress by activating NADPH oxidase to form ROS in cardiac and renal tissues, which leads to inflammation, promotes inflammatory responses and neurohormonal activation (75), and thus exacerbates heart and kidney failure. Similarly, sympathetic nervous system activation in patients with CRS leads to vasoconstriction, cardiac oxidative stress, water, and sodium retention, and release of renin, which in turn causes the release of Ang II, creating a vicious cycle. Therefore, oxidative stress is the main pathological mechanism of CRS.

Ang II is a potent inducer of ROS generation, as well as SGLT1 and SGLT2 protein expression in endothelial cells. An animal study has shown (76) that continuous infusion of Ang II leads to a significant increase in SGLT2 mRNA and protein expression in renal tissues, as well as a significant increase in ROS. This process is mediated by oxidative stress, and this response ultimately leads to endothelial aging and dysfunction. SGLT1 and SGLT2 have a decisive role in Ang II/AT1R/NADPH oxidative enzymatic stimulation signaling, which can be eliminated by SGLT2i (77). Therefore, the main mechanism of SGLT2i inhibition of oxidative stress may be the suppression of Ang II mediated oxidative stress by inhibiting Ang II induced SGLT2 overexpression and thus reducing ROS production.

Rat proximal renal tubular epithelial cells were cultured, and dapagliflozin and insulin interventions were performed in a high glucose medium environment. High glucose medium increased ROS production. While dagliflozin significantly reduced ROS production by inhibiting SGLT2 in a dose-dependent manner and also reduced intracellular ROS, IL-8, and TGF-β levels in renal tubular epithelial cells due to high glucose conditions as well as on apoptosis exerting a protective effect. This suggests that dagliflozin could inhibit oxidative stress by reducing ROS production, exerting its protective effect against cell damage, and ameliorating renal tubular oxidative stress and inflammation (78, 79). The addition of H2O2 to Human Kidney-2 mimicked the oxidative stress environment to induce cellular injury, and the production of mitochondrial ROS in the cells was found to be enhanced. Followed by intervention with dagliflozin, it was found that 0.1μM of dapagliflozin showed the greatest protective effect against H2O2-induced inhibition of cell proliferation and a significant reduction in ROS production in the mitochondria, reflecting the preventive effect and antioxidant properties of dapagliflozin on mitochondrial ROS production (80).

## 7 Conclusion

In all subtypes of CRS, the pathological mechanisms are all related to hemodynamic changes, neurohormonal mechanisms, inflammatory reactions, oxidative stress mechanisms, etc. (81). In addition, SGLT2i also has beneficial metabolic effects on failed myocardial cells, reversing myocardial remodeling, reducing proteinuria, and participating in the prevention and correction of anemia (82–85). Therefore, SGLT2i has potential therapeutic benefits for 5 types of CRS.

In this review, we focus on the therapeutic mechanism of SGLT2i acting on renal tubules for type 2 CRS. The renal tubules are the earliest and most vulnerable part of the kidney in the development of CRS, and prevention of tubular lesions is an important direction to prevent and delay the progression of CRS. In this review, we summarize the protective mechanism of SGLT2i against renal tubules. As mentioned above, SGLT2i can restore TGF, inhibit NHE3 protein expression, reduce tubular pressure and improve renal venous congestion, inhibit the expression of FGF23 and MMPs to alleviate tubulointerstitial fibrosis, regulate cellular AMP(ADP)/ATP content to promote AMPK activation to improve energy metabolism, inhibit excessive activation of neurohormones to regulate the inflammatory response, as well as inhibit SGLT2 to reduce ROS production to inhibit oxidative stress. This has profound implications for the treatment of CRS.

## Author contributions

YW conceived and wrote this article. XM was responsible for literature screening and translation. YW and XM contributed equally, together as the first author. SS, XX, and JL assisted in the literature search and data collection. HW and BZ had an outstanding contribution in second-time revision, polishing the manuscript. QS guided the design of this article. All authors contributed to the article and approved the submitted version.
